# Overcoming gaps: regional collaborative to optimize capacity management and predict length of stay of patients admitted with COVID-19

**DOI:** 10.1093/jamiaopen/ooab055

**Published:** 2021-07-08

**Authors:** Michael G Usher, Roshan Tourani, Gyorgy Simon, Christopher Tignanelli, Bryan Jarabek, Craig E Strauss, Stephen C Waring, Niall A M Klyn, Burke T Kealey, Rabindra Tambyraja, Deepti Pandita, Karyn D Baum

**Affiliations:** 1 Division of General Internal Medicine, Department of Medicine, University of Minnesota Medical School, Minneapolis, Minnesota, USA; 2 Department of Medicine, Institute for Health Informatics, University of Minnesota Medical School, Minneapolis, Minnesota, USA; 3 Division of Acute Care Surgery, Department of Surgery, University of Minnesota Medical School, Minneapolis, Minnesota, USA; 4 Department of Informatics, M Health Fairview, Minneapolis, Minnesota, USA; 5 Minneapolis Heart Institute Center for Healthcare Delivery Innovation, Minneapolis Heart Institute, Allina Health, Minneapolis, Minnesota, USA; 6 Essentia Institute of Rural Health, Essential Health, Duluth, Minnesota, USA; 7 Information Services, Essentia Health, Duluth, Minnesota, USA; 8 Internal Medicine, HealthPartners, St. Paul, Minnesota, USA; 9 Children’s Hospitals and Clinics of Minnesota, Minneapolis, Minnesota, USA; 10 Department of Medicine, Hennepin Healthcare, Minneapolis, Minnesota, USA

**Keywords:** COVID-19, Interoperability, Predictive Modeling

## Abstract

**Objective:**

Ensuring an efficient response to COVID-19 requires a degree of inter-system coordination and capacity management coupled with an accurate assessment of hospital utilization including length of stay (LOS). We aimed to establish optimal practices in inter-system data sharing and LOS modeling to support patient care and regional hospital operations.

**Materials and Methods:**

We completed a retrospective observational study of patients admitted with COVID-19 followed by 12-week prospective validation, involving 36 hospitals covering the upper Midwest. We developed a method for sharing de-identified patient data across systems for analysis. From this, we compared 3 approaches, generalized linear model (GLM) and random forest (RF), and aggregated system level averages to identify features associated with LOS. We compared model performance by area under the ROC curve (AUROC).

**Results:**

A total of 2068 patients were included and used for model derivation and 597 patients for validation. LOS overall had a median of 5.0 days and mean of 8.2 days. Consistent predictors of LOS included age, critical illness, oxygen requirement, weight loss, and nursing home admission. In the validation cohort, the RF model (AUROC 0.890) and GLM model (AUROC 0.864) achieved good to excellent prediction of LOS, but only marginally better than system averages in practice.

**Conclusion:**

Regional sharing of patient data allowed for effective prediction of LOS across systems; however, this only provided marginal improvement over hospital averages at the aggregate level. A federated approach of sharing aggregated system capacity and average LOS will likely allow for effective capacity management at the regional level.

## INTRODUCTION

Since community transmission began, exponential spread of COVID-19 has strained healthcare systems worldwide.^1–3^ While United States case numbers and hospitalizations weather multiple case fluctuations, hospitals and systems have had to undergo rapid transformation: halting or postponing elective procedures,^4^ shifting care from clinics to virtual health,^5^^,^^6^ and flexing staffing patterns to match caseloads.^2^ These dramatic changes have taken a toll on hospital-based care delivery, both financially and on providers and staff.^7^^,^^8^ Patient access to acute care and the quality of care delivery during periods of strain have been significant challenges related to COVID-19 and will continue even as the pandemic wanes.

Ensuring an efficient response to patient surges including COVID-19 requires a degree of coordination and capacity management across healthcare systems that has not existed in the US healthcare. Cohorting patients, whether for COVID-19 care, thereby keeping COVID-19 infected patients away from the general inpatient population to reduce nosocomial transmission, or other subspecialty care has the potential to improve delivery. Unfortunately, flexible bed availability is not guaranteed to be equal across all hospitals, particularly in small rural hospitals and safety net hospitals.^9^

Efficient capacity management requires several pieces of information: a predicted number of new admissions, an accurate accounting of hospital and intensive care unit (ICU) beds and staffing levels, and a prediction of the number of discharges. If the number of new cases exceeds the number of discharges, increasing staffing, and reducing avoidable admissions such as elective procedures becomes necessary. If the number of predicted discharges exceeds the number of predicted cases, these changes can be relaxed. Predicting the number of discharges requires an accurate understanding of inpatient length of stay (LOS).

Inefficient planning for patient surges has substantial cost. Over-preparing could result in unnecessary delay for non-emergent, but time-sensitive procedures and delayed inpatient care, as well as avoidance of hospitals by patients when emergent care is necessary.^10^^,^^11^ Overstaffing in the short term could result in future staffing shortfalls, and an overall extended financial loss of hospital systems. Conversely, under-staffing in anticipation of patient surges can adversely impact the quality of patient care. Strain related to high patient volume has been associated with higher costs, worse outcomes, and provider burnout.^12–15^

Finally, given hot spots in transmission, single hospital systems may become overwhelmed, exceeding its ability to safely care for patients. Thus, single health care systems in isolation are will inevitably respond to patient surges inefficiently; regional level capacity management is a needed component to an efficient pandemic response.^16^ A successful program requires a degree of regional data sharing of current case load, capacity and projected capacity as well as coordination of beds and staffing including safe inter-hospital transfers to ensure level loading of work. While electronic health record interoperability solutions provide access to patient level data across health systems, evidence to support its use for capacity management and hospital operations is lacking. Taken together an optimal program to ensure efficient hospital-based care for COVID requires addressing 2 critical gaps in the literature: predicting LOS after admission, thereby predicting the number of discharges, and sharing that data across systems.

Even now, despite over a year of experience with COVID-19, limited published data exist on predicting hospital LOS for COVID-19 patients. Many published studies of hospitalized COVID-19 patients occurred outside the United States, or during periods of strain, limiting generalization.^17^^,^^18^ A meta-analysis of COVID-19 LOS demonstrated wide variability in reported hospital utilization.^19^ While models have been developed to identify patients at high risk for mortality, studies illustrating variation and factors associated with LOS are lacking.^20–22^ Predictors of risk of death may overlap with LOS, but LOS models may also contain unique predictors that describe the clinical course of patients with this novel illness. Prediction of LOS serves 2 potential purposes: to inform direct patient care and to inform the health system of future resource needs. Implementing models that facilitate coordination of healthcare at the population level is an important consideration in developing a regionally coordinated surge plan.^23^

Approaches to coordinating hospital operations could follow a federated or centralized model. In a federated approach, a predictive model would be deployed at each site, and aggregated data shared together. In a centralized approach, patient level data could be shared and analyzed at one site, allowing more complex modeling approaches including machine learning and support health systems with informatics resource constraints.^24^^,^^25^ In this study, we describe the development of a multi-system regional collaborative to improve regional hospital capacity management during the COVID-19 pandemic. We demonstrate an effective way to share patient information to support both hospital operations and research. Finally, we compare approaches in the prediction hospital LOS to support care at the patient and system level to identify best practices.

## MATERIALS AND METHODS

### Setting

Supported by the Minnesota Hospital Association, we developed a multi-system collaborative covering the states of Minnesota, Wisconsin, and the Dakotas, with 36 hospitals caring for approximately 60% of the state of Minnesota’s hospitalized COVID-19 population. Chief Medical Informatics Officers (CMIOs) and other stakeholders from each health system meet virtually on a weekly basis. Issues surrounding bed capacity, informatics needs during the pandemic, and general information regarding LOS, were shared within the group. The group also utilized a common model to help systems predict future cases. As part of this effort, the committee undertook a collaboration to determine LOS statistics, as well as predictors of LOS for individual patients. This effort was determined to be exempt by the Institutional Review Board of each participating organization.

### Patients

De-identified patient data using a safe harbor approach according to institutional protocols was shared between each participating hospital system using a HIPAA secure file sharing service (Box; Box, Inc.).^25^ Patients admitted between March 13 and June 12, 2020 were included if they tested positive by PCR for SARS-CoV-2 either during their stay or in the 2 weeks prior to their admission. Data were abstracted from multiple electronic medical records. The validation cohort was drawn from consecutive COVID-19 admissions from a 12-hospital subsample from June 12th and discharged by September 14th.

### Measures

Standardized definitions were developed across health systems including ICU LOS, ventilator days, and comorbidities (Supplemental digital content for protocol). We obtained patient demographics including age and race, admission body mass index, first set of vitals, ventilator, and ICU utilization, whether the patient underwent an inter-hospital transfer, and whether the patient was admitted from a nursing facility. All ICD codes in the year prior to admission were extracted and converted into chronic comorbidities following Elixhauser.^26^ We used median imputation to adjust for missing variables. If patients lacked ICD coding for a given chronic diagnosis this was considered to be negative.

### Model derivation

We compared 2 approaches to predict LOS based on ease of implementation. First, we used a multivariate generalized linear model (GLM) with discharge by 5, 10, and 15 days as the dependent variable, including random effects for each hospital system. We included comorbidities, initial vitals, age, race, need for ICU or ventilator support, maximum O_2_ requirement, nursing home admission, and inter-hospital transfer as initial potential predictors. Final model features were selected using PC-Simple with maximum condition set size of 3.^27^

The LOS prediction from the regression model was compared against a predictive model generated by a random forest (RF), which reduces potential bias from errors in assumption regarding the relationship and interaction of factors.^28^^,^^29^ For each RF model, we generated variable importance plots by Gini impurity index. For simplicity, we report only the top 20 variables by importance. We used area under the ROC curve (AUROC) to compare accuracy of both models. For each, we used test sets of 5-fold cross-validation with 95% confidence intervals (CIs) calculated by 200 bootstrap samples. AUROC for both models were compared for 5, 10, and 15-day thresholds as well as mortality. Calibration curves for each model are provided in the Supplementary text.

### Validation

To simulate effectiveness of LOS modeling to predict capacity at a healthcare system level we performed 2 separate analysis to simulate weekly meetings of the CMIO workgroup. Using a 12-week validation cohort of consecutive admissions to a 12-hospital subsample, we split patients into weekly cohorts flagged by whether they were discharged or still admitted on Friday of that week. We allowed the model to be recalibrated weekly with additional discharges to adjust for time-sensitive confounders. For patients still admitted we tested the ability of each model to predict the likelihood that each individual still admitted would be discharged at 5, 10, and 15 days. We compared performance against what would be predicted by the unadjusted population average for that health system to simulate a federated model with only summary data.

### Statistics

We first illustrate the population and association with LOS via Mann–Whitney tests. A Bonferroni correction was added to adjust for multiple hypothesis/comparison testing. Paired *t*-test was used to compare AUROCs across 12 weeks of validation. Stata (v14) and R were used for all statistical analyses.

## RESULTS

A total of 2068 patients were admitted to one of 36 hospitals during the study period. Within the cohort, a majority of patients were non-White, and nearly a quarter (24%) were older than 75. A majority (77.4%) of patients had at least one chronic illness. Nearly one quarter of admitted patients required care in an ICU, with 16.6% requiring mechanical ventilation (Table 1).

**Table 1.** Demographics, comorbidities, and complications of COVID-19 and univariate association with length of stay

	Derivation			Validation		
	*n* (%)	Length of stay, days (median, IQR)	*P*-value	*n* (%)	Length of stay, days (median, IQR)	*P*-value
Total	2068	5 (2.34, 9.84)		597^a^	5.13 (2.46, 9.94)	
Age (years)						
0–5	27 (1.3%)	2 (1.0, 4.0)	<.001	3 (0.5%)	2.2 (2.1, 8.0)	.434
5–18	24 (1.2%)	2 (1.0, 4.0)	<.001	5 (0.8%)	4.9 (3.4, 11.1)	.766
18–35	213 (10.3%)	2.8 (1.8, 5.8)	<.001	99 (16.6%)	2.1 (1.4, 3.7)	<.001
35–55	505 (24.4%)	4.13 (2.0, 7.6)	<.001	134 (22.4%)	5.9 (2.6, 10.0)	.534
55–75	789 (38.2%)	5.87 (2.9, 11.2)	<.001	223 (37.3%)	6.1 (3.4, 10.9)	<.001
Greater than 75	510 (24.7%)	6 (3.3, 11.0)	<.001	133 (22.2%)	6.7 (3.6, 11.7)	<.001
Male	1006 (48.6%)	5 (2.3, 10.1)	.773	282 (47.2%)	6 (3.0, 12.9)	<.001
Race						
White	933 (48.1%)	5.86 (3.0, 11.0)	<.001	285 (47.7%)	5.1 (2.7, 9.1)	.890
Black	475 (23.0%)	4.32 (2.1, 8.5)	.052	119 (5.3%)	3.7 (2.1, 8.7)	.039
Hispanic	260 (12.6%)	4 (2.0, 7.8)	.001	32 (5.4%)	6.1 (2.4, 11.0)	.573
Asian	172 (8.3%)	4.7 (2.2, 9.5)	.597	77 (12.9%)	5.8 (2.5, 12.6)	.171
Native American	67 (3.2%)	4 (2.26, 7)	.172	11 (1.8%)	5.2 (2.7, 9.3)	.341
Other/missing	184 (8.9%)	4 (2.0, 9.0)	.015	64 (10.7%)	6.1 (3.1, 11.7)	.083
Comorbid conditions						
No comorbidities	468 (22.6%)	4 (2.0, 8.0)	<.001	63 (10.6%)	2.6 (1.6, 5.1)	<.001
Hypertension	899 (43.4%)	5.9 (3.0, 11.6)	<.001	363 (60.8%)	6.2 (3.6, 11.8)	<.001
Diabetes	213 (10.3%)	7.73 (2.9, 11.1)	<.001	227 (38.0%)	6.1 (3.4, 11.6)	<.001
Obesity	1355 (65.6%)	5.15 (2.5, 10.2)	.001	457 (76.5%)	6.0 (2.9, 11.4)	<.001
Chronic kidney disease	360 (17.4%)	7.67 (3.7, 13.0)	<.001	166 (27.8%)	7.3 (4.3, 14.3)	<.001
Chronic obstructive pulmonary disease	372 (18.0%)	5.5 (2.8, 10.6)	.054	147 (24.6%)	6.1 (3.2, 11.0)	.026
Cancer	129 (6.2%)	6.3 (3.37, 12.0)	.011	69 (11.6%)	5.1 (3.0, 9.2)	.555
Congestive heart failure	255 (12.4%)	6.72 (3.7, 12.2)	<.001	128 (21.4%)	7.1 (3.9, 12.2)	<.001
Critical illness						
General floor	1645 (79.6%)	4.1 (2.0, 7.5)	<.001	344 (57.6%)	3.2 (2.0, 6.2)	<.001
ICU without mechanical ventilation	84 (4.0%)	7.9 (4.7, 11.4)	<.001	184 (30.8%)	6.9 (5.0, 11.1)	<.001
ICU with mechanical ventilation	339 (16.4%)	14 (6.7, 23.9)	<.001	69 (11.6%)	18.1 (12.5, 30.7)	<.001
Patient flow						
Admit from a nursing facility	200 (9.7%)	9 (5, 15.9)	<.001	72 (12.1%)	7.7 (4.4, 11.8)	<.001
Inter-hospital transfer	378 (18.3%)	8.6 (4.3, 15.9)	<.001	336 (56.2%)	7.4 (5.0, 13.6)	<.001

*Note*: Threshold for statistical significance <0.002.

^a^Includes 40 patients who were admitted during the validation period but were discharged afterwards. These were not included in the week to week validation analysis.

Overall LOS followed a long-tailed distribution with a mean of 8.2 days and median of 5.0 days and an interquartile range (IQR) of 3 days. Pediatric cases (<18 years old) made up only 3% of the cohort and had an overall shorter LOS than the general population (*P* < .0001). Patients who required mechanical ventilation required markedly longer LOS with a median of 14.0 inpatient days (IQR 6.7–23.9) when compared with patients who were admitted to an ICU but did not require intubation (8 days, IQR 7.9 [4.7 to 11.4]) or those who remained on a general floor (median 4.1, IQR 2 to 7.5, *P* < .001). Similarly, chronic comorbidities including hypertension, diabetes mellitus, chronic kidney disease, and congestive heart failure were associated with longer LOS (*P* < .001).

**Figure 1.**
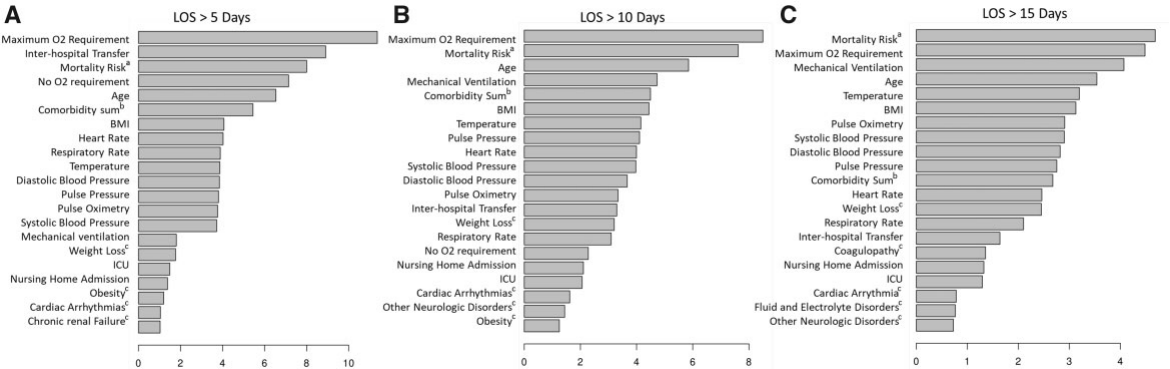
Multivariate prediction of hospital length of stay. Patient predictors by importance (approximated by the Gini impurity index) for length of stay >5 days (A), AUC 0788 (95% CI 0.732–0.784). Length of stay >10 days (B), AUC 0.814 (95% CI 0.751–0.807), and length of stay >15 days (C), AUC 0.836 (0.801–0.860). ^a^Mortality risk: independent risk of mortality, generated by a random forest model excluding complications illustrated in Supplementary Figure S2. ^b^Elixhauser comorbidity sum. ^c^From individual elixhauser comorbidities. All vitals represent the first vital taken that admission.

Overall multivariate prediction via GLM achieved fair to good prediction of mortality and LOS at 5 (AUROC 0.772 95% CI 0.732–0.784), 10 (AUROC 0.778 [0.751–0.807]) and 15 days (AUROC 0.800 [95% CI 0.766–0.828]), Supplementary Appendix Table S1). The RF models slightly outperformed the GLM models and achieved fair to good discrimination (LOS > 5 days: AUROC, LOS > 10 days: AUROC 0.801, LOS > 15 days: AUROC 0.836, Supplementary Appendix Table S1 and Figure S1). Predictors of LOS which were consistent across LOS thresholds and approaches included age, admission from a nursing home, Critical illness and mechanical ventilation, maximum O_2_ requirement, and weight loss (Figure 1, Table 2). Features associated with in-hospital mortality significantly overlapped with those predicting LOS (Supplementary Appendix Table S2 and Figure S2).

**Table 2.** Multivariate prediction of hospital length of stay by a generalized linear model

	LOS >5 days (AUROC 0.772: 95% CI 0.732–0.784)			LOS >10 days (AUROC 0.778: 95% CI 0.751–0.807)			LOS >15 days (AUROC 0.800 95% CI 0.766–0.828)		
	Coef	Standard error	*P*-value	Coef	Standard error	*P*-value	Coef	Standard error	*P*-value
Age	0.012	0.054	<.001	0.012	0.054	<.001	–	–	NS
Admission from nursing home	1.028	0.057	<.001	1.028	0.057	<.001	–	–	NS
Inter-hospital transfer	0.495	0.055	<.001	0.495	0.055	<.001	–	–	NS
ICU	0.958	0.053	<.001	0.958	0.053	<.001	0.793	0.104	<.001
No O_2_ administered	−1.361	0.054	<.001	−1.361	0.054	<.001	−0.811	0.102	<.001
Weight loss	0.723	0.060	<.001	0.723	0.060	<.001	0.653	0.061	.001
Mechanical ventilation	–	–	NS	–	–	NS	1.418	0.089	<.001
Coagulopathy	–	–	NS	–	–	NS	0.697	0.058	.002

**Figure 2.**
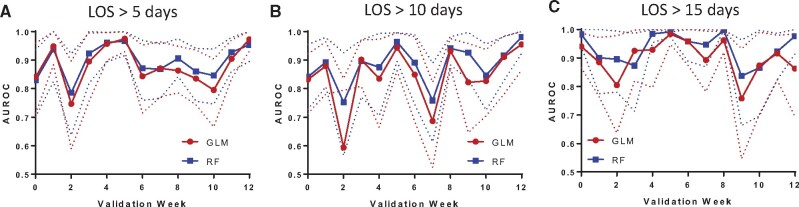
Comparison of weekly model performance between generalized linear model (GLM) and random forest (RF) models across a 12-week validation period measured by AUROC (solid line) and 95% CI (dotted lines for upper and lower limits). A. Length of Stay > 5 days, B. Length of Stay > 10 days, C. Length of Stay > 15 days.

Week to week validation over a 12-week period is displayed in Figure 2 (Supplementary Appendix Figures S3–S6). Week to week variation in LOS is illustrated in Supplementary Appendix Figure S7. The RF outperformed GLM model in prediction as measured by AUROC (Figure 2). Paired *T*-test comparing the 2 models demonstrated statistical differences for LOS >5 days (*P* = .012), LOS >10 days (*P* = .042) and LOS >15 days (*P* = .038). However, both achieved good to excellent accuracy on a week to week basis.

Despite week to week recalibration of the LOS model, predictors remained durable over time. Predictors including oxygen requirement, age, nursing home admission, inter-hospital transfer, critical illness, and weight loss being consistently selected as important by both RF and GLM models across the duration of the study (Supplementary Appendix Figures S8 and S9, Tables S3 and S4).

Each model including the aggregated model (approximating a federated model of data sharing), were effective in predicting the number of patients that were would be discharged by 5, 10, and 15 days (Table 3). Overall, the RF model outperformed the other 2 approaches, accurately predicting the number of discharges at all-time points for greater than 90% of cases 8 out of the 12 weeks. However, in aggregate relying on the unadjusted average to predict the following week performed nearly as well particularly at 5-day and 15-day thresholds.

**Table 3.** Aggregate performance of random forest (RF) and generalized linear model (GLM) against the unadjusted population average (Avg) in predicting future discharge timing for patients admitted during a 12-week validation period.

		Discharged in 5 days				Discharged in 10 days				Discharged in 15 days			
		Predicted			Actual	Predicted			Actual	Predicted			Actual
Week	*n*	RF	GLM	Avg		RF	GLM	Avg		RF	GLM	Avg	
1	42	14	14	17	14	21	21	28	20	26	26	34	27
2	34	11	14	13	10	17	15	22	17	19	22	27	23
3	33	16	15	15	15	22	21	22	22	25	26	26	26
4	40	13	21	16	15	22	20	25	20	33	34	32	30
5	38	17	21	15	12	24	27	24	26	27	27	30	29
6	51	27	21	20	33	38	35	41	39	42	41	40	43
7	38	14	11	15	21	28	26	25	32	32	32	30	36
8	46	24	23	19	21	39	36	30	36	41	42	37	42
9	70	28	29	29	28	51	47	46	47	58	54	56	57
10	59	21	18	24	23	44	43	39	46	51	51	48	49
11	48	22	18	20	19	35	37	32	31	44	40	39	40
12	58	27	28	24	30	45	58	39	44	52	45	47	49
Total:	557	234	233	227	241	386	386	373	380	450	440	446	451

## DISCUSSION

The COVID-19 pandemic has exposed many weaknesses of the US healthcare system including inequities in access, gaps in coordinated testing, partial insurance coverage, shortages in PPE supply and distribution, inefficiencies in data aggregation, challenges with dissemination of trends to the general public, inconsistent public health policy, and inconsistent public health communication. A common thread which ties these flaws together is fragmentation.^30–32^ Overcoming these challenges requires a degree of coordination across health systems which has not existed throughout the history of American hospital-based care. In this study, we try to overcome fragmentation by centralizing data sharing using a common governance board for the purposes of capacity management, population health, and research. Most importantly, we demonstrate that while many of the barriers to population management of COVID-19 can be overcome, but a federated approach will likely be as successful to support future surges.

While electronic health record adoption has reached near ubiquity in hospitals, interoperability between health systems has lagged. While solutions such as Epic Care Everywhere have progressed, the primary goal of these efforts is to improve care at the level of an individual patient. As a result, these solutions are gated by informed consent requiring patient presence, and thus cannot be used across systems for population health. Instead, we relied on sharing aggregated data for capacity management and a standardized data collection and de-identification process. De-identification allowed us to share patient level information across systems for analysis while minimizing risk to patient confidentiality.^25^ Overcoming these barriers was effort intense, requiring many man-hours of validation and processing to ensure uniformity.

We then tested various approaches to prediction to optimally guide patient care and capacity planning. We found the RF model provided good to excellent prediction of LOS at both the patient and aggregate level, and provided a slight improvement over the GLM model. Consistent predictors of LOS separate from mortality included weight loss likely indicating frailty, nursing home admission, and illness severity defined by vitals, oxygen requirement, and critical illness. In the optimal setting, widely distributed machine learning algorithms could provide near real-time updates and ensure accurate projection of occupied hospital beds, informing patients of their likely hospital course and leadership of capacity needs. At a minimum, LOS is likely to vary significantly by regional demographics: populations with greater proportions of elderly with a higher burden of chronic illness such as observed in rural areas will likely have disproportionately greater hospital utilization that warrants added preparation. These predictors are likely not unique to COVID-19.

The included cohort was majority non-White, markedly different than state demographics. However, while race was included as a potential feature, it was not a statistically significant predictor of LOS in multivariate analysis. These findings are consistent with published literature that racial disparities in COVID-19 are caused primarily by higher transmission rates, and subsequent differences in outcomes are largely explained by comorbidity rates and testing gaps.^33^

In aggregate, while the RF model performed better than the unadjusted population average, barring any substantial shifts in the population makeup, knowing what percentage of COVID-19 patients are discharged by 5, 10, and15 days is likely sufficient to predict future utilization. Similarly, at the patient level, most of the factors identified in the predictive models are naturally self-evident and follow established clinical course.^34^ Thus a federated approach sharing aggregated data is likely sufficient for health system coordination. At the patient level predictive modeling of LOS is unlikely to substantially improve over clinical gestalt. The effort required for broad deployment of machine learning technology to regional hospital systems that lack capability or establishing consistent data sharing, verification and analysis is likely not worth the return on investment.

Our findings suggest the informatics barriers to system-to-system coordination are actually quite low. Patient level data sharing, while helpful from an exploratory standpoint, is not needed to support surge planning or hospital operations. Coordination merely requires sharing aggregated information: current total and ICU census and LOS distribution for each health system. This allows the focus of coordination to remain on surveillance and prediction of caseloads, optimal cohorting strategies and level loading across systems to adapt to capacity strain.

This approach has the potential to improve care beyond the current pandemic. Capacity strain is a common occurrence in US hospitals even prior to the spread of COVID-19. Adjusting patient distribution during other periods of high capacity could substantially improve care, ensuring patients with specialized needs have available beds at the locations they need. Regional transfer centers that support care across health systems could be an effective next step from this work.

There are several limitations of this study. First, this is an observational study from a regional cohort of only COVID-19 patients, relying on de-identified data limits this study’s generalizability to other contexts. While we identify several important predictors of LOS, studies which predict risk have shown significant variability from cohort to cohort. Establishing local trends in LOS is critically important as the observed LOS in our study deviated substantially from previously published models predicting caseloads and surge capacity.^16^ As such, we do not place substantial weight on individual predictors. The primary contribution of this work is to establish optimal approaches to data sharing and prediction to guide care at the patient and system level. Additionally, since de-identified data was used, linkage across systems was not possible, thus readmissions to other systems and transfers across systems would not be completely captured.

Second, during data collection several potential novel predictors of risk including laboratory values such as d-dimer and C-reactive protein were identified.^20^^,^^35^ While we attempted to collect this data, limitations in additional analytics and informatics support at participating healthcare systems, as well as variations in practice resulted in high rates of missing data that could not be reasonably imputed. It is likely with additional data we could further improve predictions; however, this also reflects real-world limitations in data collection particularly from community hospital systems with resource constraints.

While patient level data sharing for establishing hospital LOS is unlikely to be cost effective, that does not mean that regional data sharing cannot assist other elements of the COVID-19 pandemic or other surges in capacity. particularly in surveillance of COVID-19 cases among patients who receive care at multiple hospitals. Our effort in establishing a mechanism to share patient level data is a first step in coordination which in the future could identify patients who fall through the cracks between health systems and responding other time points where hospital capacity is strained.^30^ Overcoming obstacles such as competition across health systems would be critical for such a program’s success.

Finally, while we focus on COVID-19, this represents a minority of patients admitted to the hospital. Predicting total and future capacity requires a better understanding of hospital LOS and predicting hospitalization rate in a heterogeneous population. There may be a role for machine learning in this more heterogeneous and uncertain population supported by more granular longitudinal data that deserves to be better studied.^28^^,^^36^

## CONCLUSIONS

In this study, we examine hospital utilization of patients admitted with COVID-19 in the context of a regional collaborative to support capacity management and surge planning. We demonstrate that patient level data sharing is possible between systems and overcoming multiple electronic record systems, allowing for effective prediction of LOS. However, this process was resource intense, and likely provides limited benefit above clinical reasoning and unadjusted population averages. A federated approach to regional coordination including inter-system sharing of aggregated medicine and ICU capacity and average LOS for COVID-19 patients are likely sufficient to coordinate for patient surges.

## FUNDING

This research was supported by the Agency for Healthcare Research and Quality (AHRQ) (R01HS026732 to MGU). The content is solely the responsibility of the authors and does not necessarily represent the official views of AHRQ.

## AUTHOR CONTRIBUTION

All authors contributed to this manuscript and certify that they sufficiently participated and are responsible for the work. MGU, GS, BJ, and KDB were responsible for overall concept and design. MGU, RT, and GS were responsible for validation and statistical analysis. All authors were responsible for critical revision including intellectual content and all were involved in data acquisition. KDB and BJ supervised the project.

## SUPPLEMENTARY MATERIAL

Supplementary material is available at *Journal of the American Medical Informatics Association* online.

## Supplementary Material

ooab055_Supplementary_Data
