# Supplementary material for: Overcoming gaps: regional collaborative to optimize capacity management and predict length of stay of patients admitted with COVID-19
Source: JAMIA Open. 2021 Jul 8;4(3):ooab055. doi: 10.1093/jamiaopen/ooab055 (PMC8327377; doi:10.1093/jamiaopen/ooab055)
Supplement: ooab055_Supplementary_Data [file ooab055_supplementary_data.docx]

Appendix Table of Contents:

**Figure 1: Receiver Operator curves for various length of stay thresholds comparing a generalized linear model and random forest approaches.**

**Table 1: Summary of AUROC with 95 % CI of all models included in the study.**

**Table III: Selected factors predicting in-hospital mortality by GLM model**

**Figure 2: Top 20 factors by variable importance (approximated by the Gini impurity index) associated with in-hospital mortality selected by the random forest model.**

**Figure 3: Receiver operator curves for GLM model predicting LOS at 5,10 and 15 day thresholds including validation cohort.**

**Figure 4. Receiver operator curves for RF model predicting LOS at 5,10 and 15 day thresholds including validation cohort.**

**Figure 5: Calibration curves of GLM models predicting length of stay at 5, 10, and 15 day thresholds.**

**Figure 6: Calibration curves of RF models predicting length of stay at 5, 10, and 15 day thresholds.**

**Figure 7: Weekly distribution of length of stay during the validation period.**

**Figure 8: Durability of factors associated with LOS > 5 days identified by a random forest following recalibration at weeks 1, 4, 8, and 12.**

**Figure 9: Durability of factors associated with LOS > 10 days identified by a random forest following recalibration at weeks 1, 4, 8, and 12.**

**Table 4: Durability of factors associated with LOS > 5 days by GLM following recalibration at weeks 1,4,8,12**

**Table 5: Durability of factors associated with LOS > 10 days by GLM following recalibration at weeks 1,4,8,12**

**Table 6: Durability of factors associated with LOS > 15 days by GLM following recalibration at weeks 1,4,8,12**

**Figure 1: Receiver Operator curves for various length of stay thresholds comparing a generalized linear model and random forest approaches.**


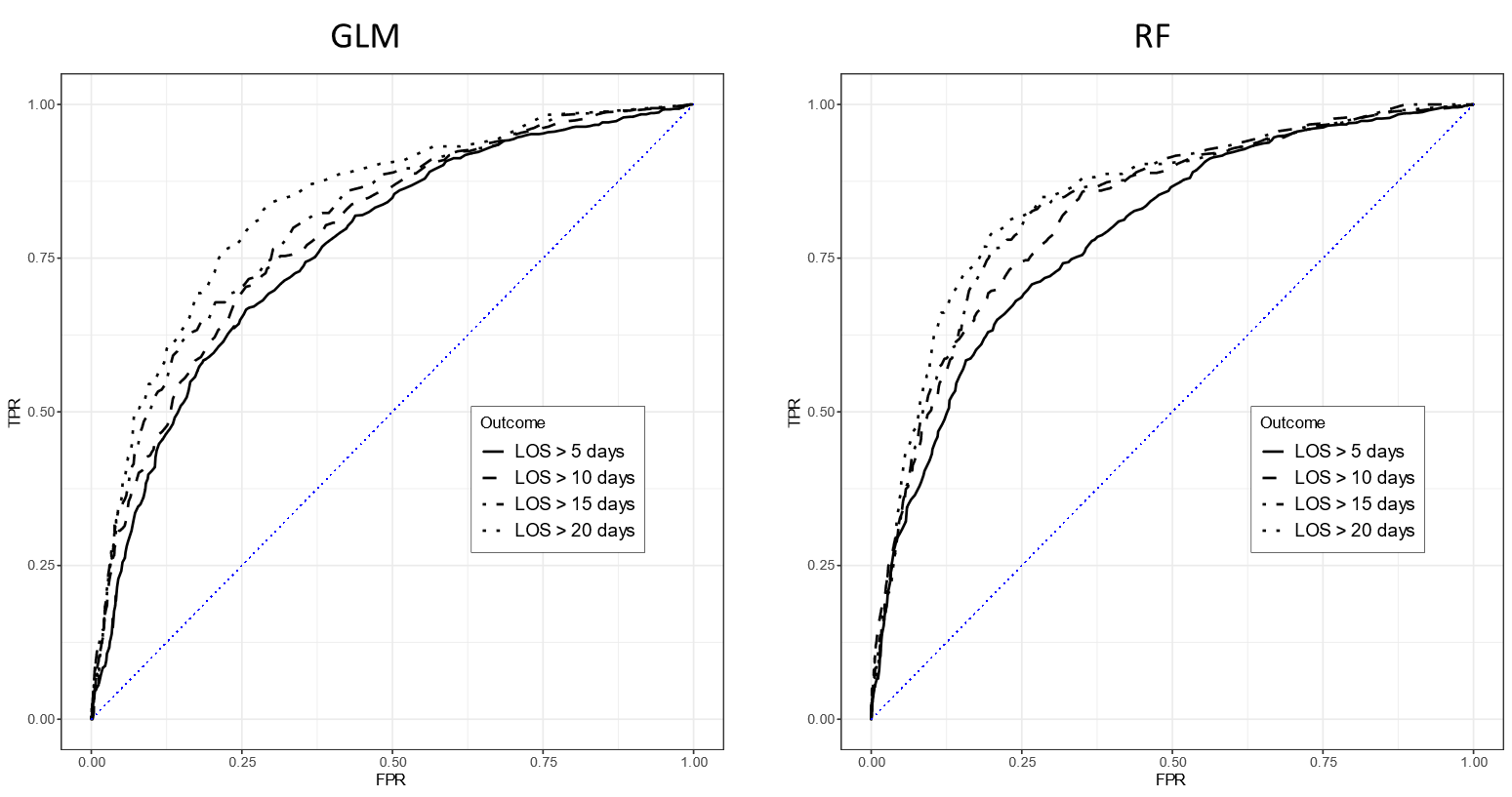


**Table 1: Summary of AUROC with 95 % CI of all models included in the study.**

|  | Derivation | | Validation | |
| --- | --- | --- | --- | --- |
|  | GLM (95% CI) | RF (95% CI) | GLM (95% CI) | RF (95% CI) |
| Inpatient Mortality | 0.862 (0.834, 0.89) | 0.878 (0.851, 0.905) | 0.855 (0.810, 0.900) | 0.890 (0.855, 0.925) |
| LOS > 5 Days | 0.772 (0.732, 0.784) | 0.788 (0.759, 0.808) | 0.864 (0.833, 0.891) | 0.890 (0.865, 0.917) |
| LOS > 10 Days | 0.778 (0.751, 0.807) | 0.814 (0.788, 0.842) | 0.835 (0.798, 0.864) | 0.877 (0.847, 0.906) |
| LOS > 15 Days | 0.800 (0.766, 0.828) | 0.836 (0.801, 0.860) | 0.868 (0.824, 0.903) | 0.910 (0.874, 0.941) |

**Table 3: Selected factors predicting in-hospital mortality by GLM model**

|  | Coefficient Estimate | Standard Error | p value |
| --- | --- | --- | --- |
| Age | 0.07 | 0.01 | <0.001 |
| Black | -1.48 | 0.33 | <0.001 |
| Nursing home Admission | 0.98 | 0.29 | 0.001 |
| ICU | 1.00 | 0.31 | 0.001 |
| Maximum O2 Requirement | 0.02 | 0.01 | <0.001 |
| Mechanical Ventilation | 1.46 | 0.32 | <0.001 |
| Hypertension with complications* | 0.65 | 0.21 | 0.002 |
| Respiratory Rate | 0.04 | 0.01 | 0.006 |
| Diastolic Blood Pressure | -0.02 | 0.01 | 0.002 |

**Figure 2: Top 20 factors by variable importance (approximated by the Gini impurity index) associated with in-hospital mortality selected by the random forest model.**

**
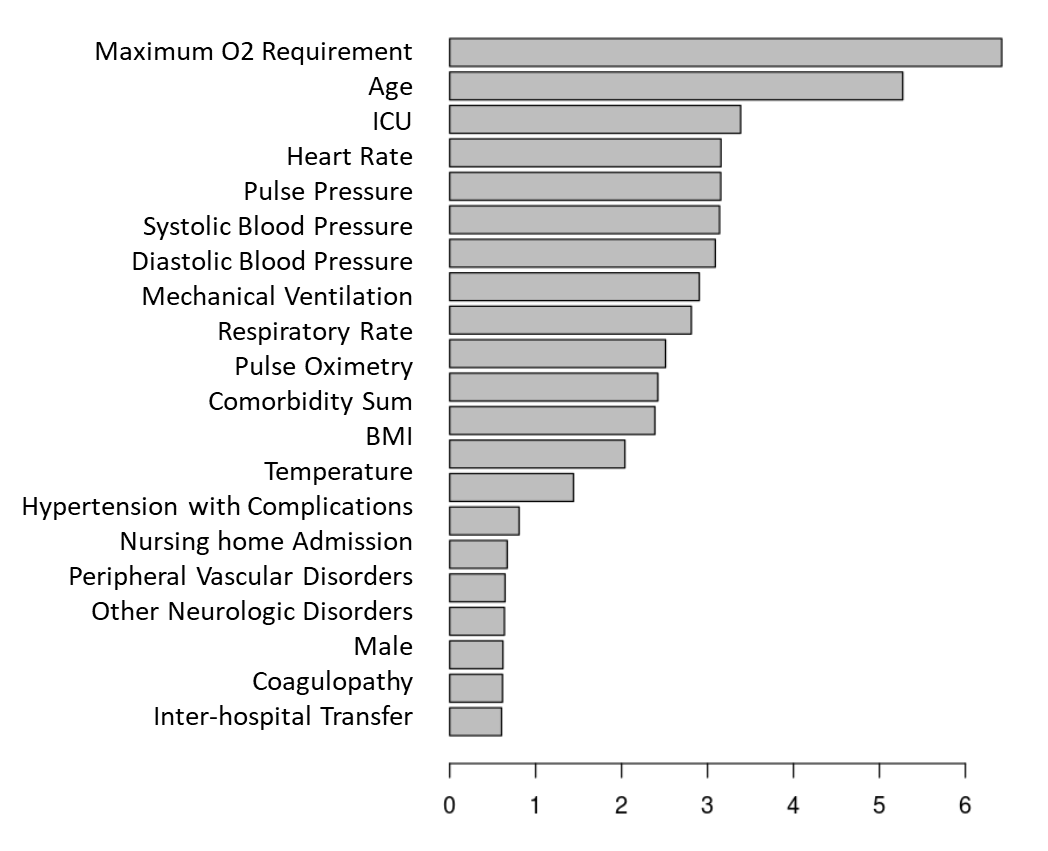
**

**Figure 3: Receiver operator curves for GLM model predicting LOS at 5,10 and 15 day thresholds including validation cohort.**


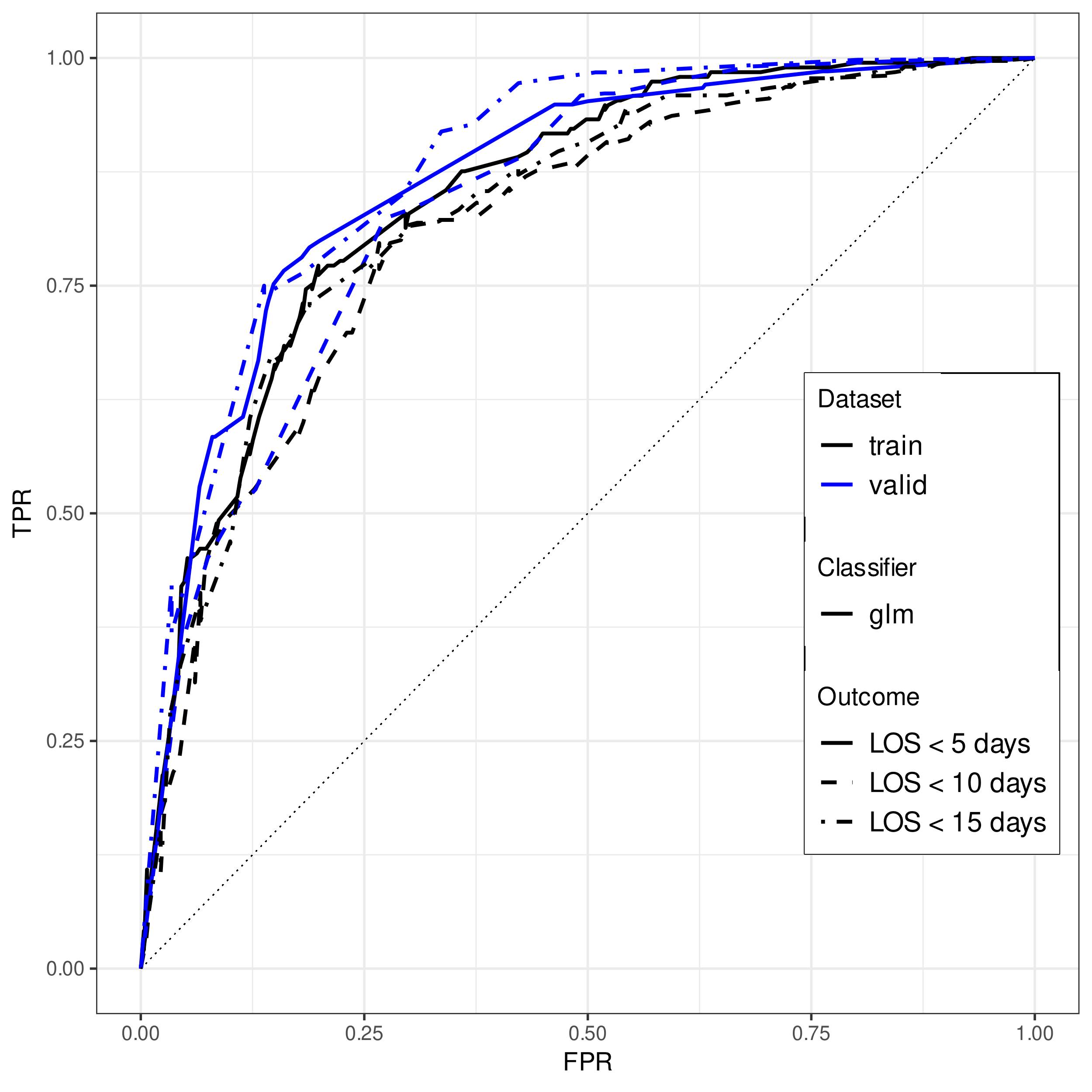


**Figure 4. Receiver operator curves for RF model predicting LOS at 5,10 and 15 day thresholds including validation cohort.**


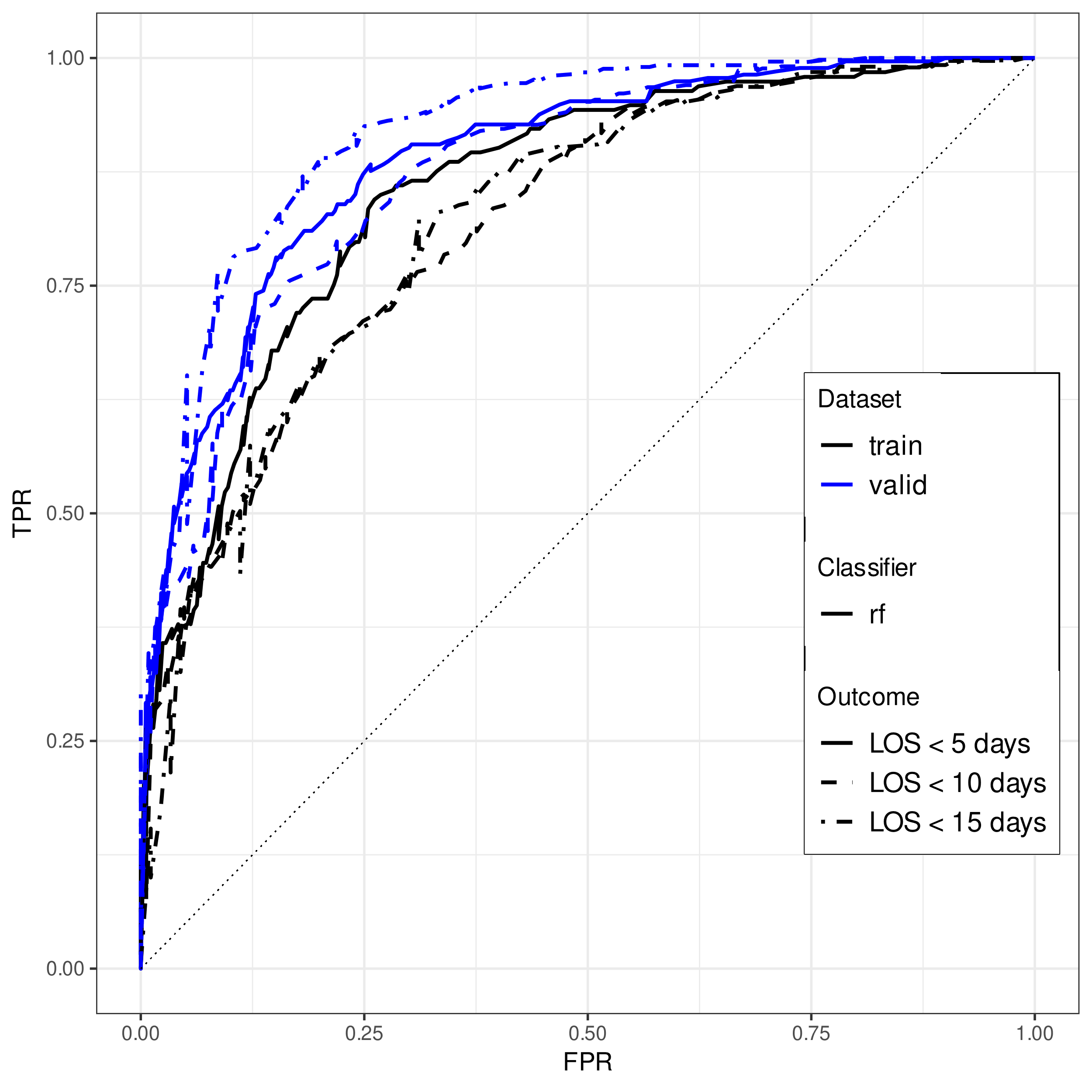


**Figure 5: Calibration curves of GLM models predicting length of stay at 5, 10, and 15 day thresholds.**


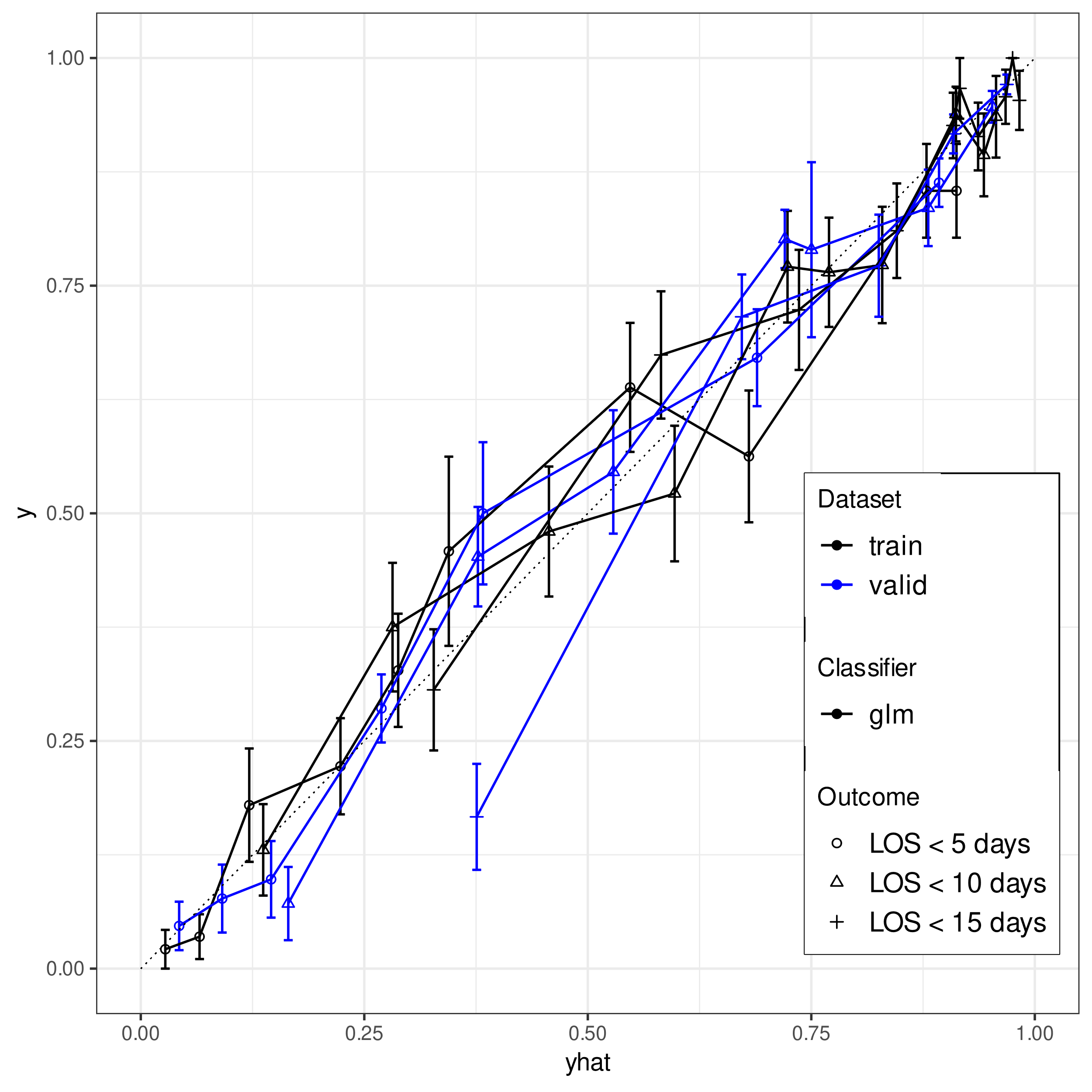


**Figure 6: Calibration curves of RF models predicting length of stay at 5, 10, and 15 day thresholds.**

**
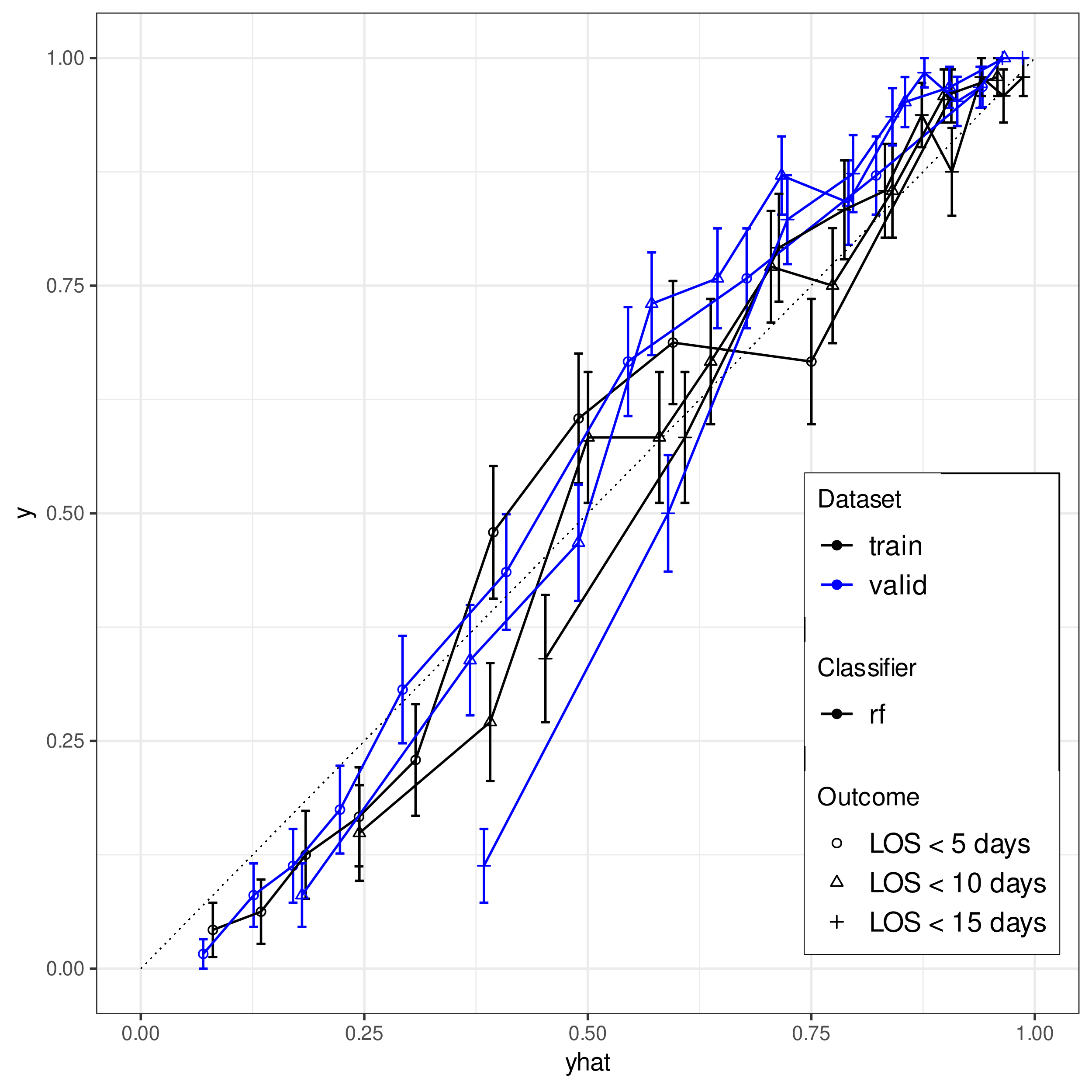
**

**Figure 7: Weekly distribution of length of stay during the validation period.**


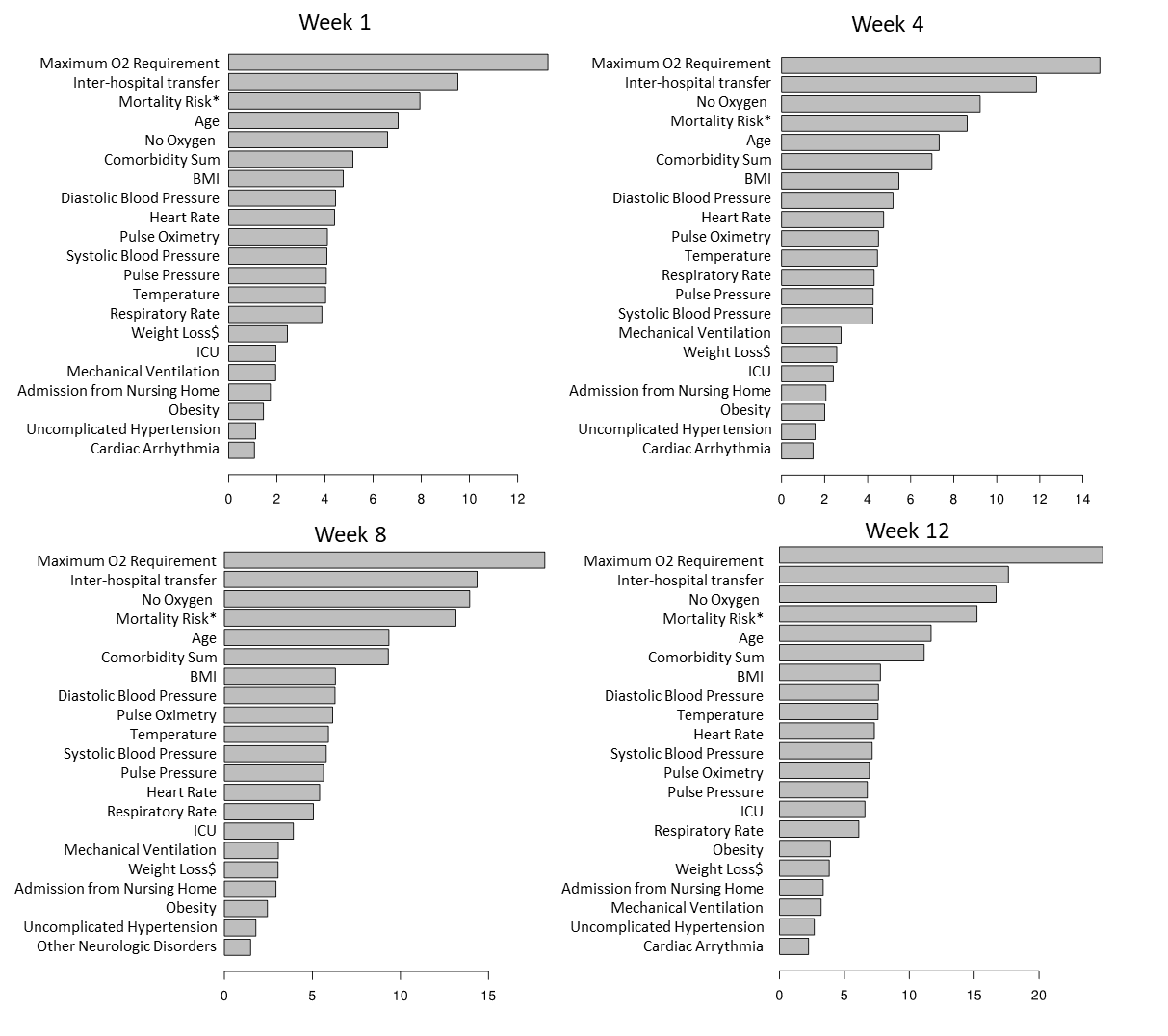


**Figure 8: Durability of factors associated with LOS > 5 days identified by a random forest following recalibration at weeks 1, 4, 8, and 12.**

* The model was allowed to recalibrate on a weekly basis including new patients that had been discharged prior to extraction (occurring on Friday) This shows selected weeks demonstrating factors remained consistent through time.


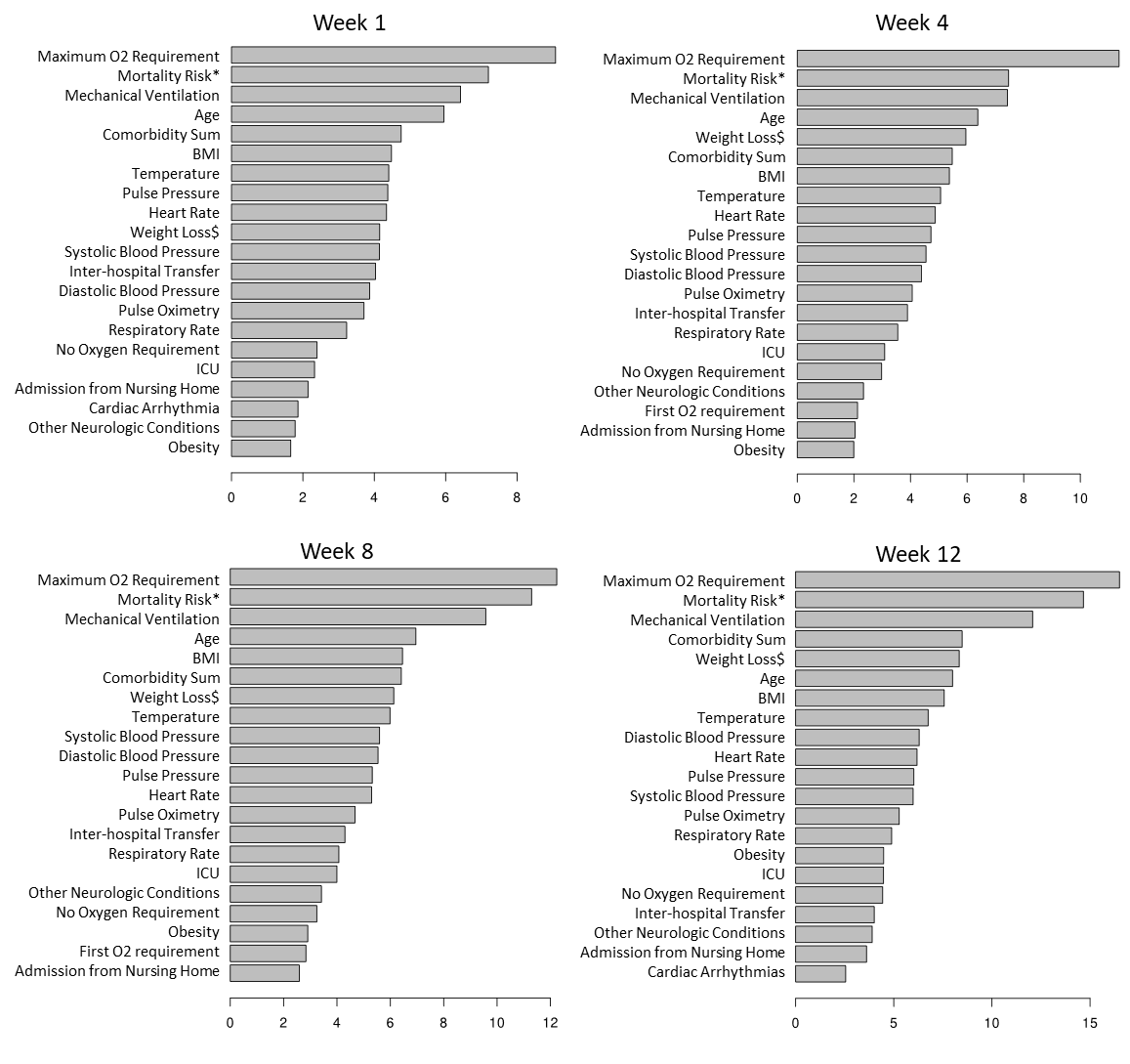


**Figure 9: Durability of factors associated with LOS > 10 days identified by a random forest following recalibration at weeks 1, 4, 8, and 12.**

* The model was allowed to recalibrate on a weekly basis including new patients that had been discharged prior to extraction (occurring on Friday) This shows selected weeks demonstrating factors remained consistent through time.

**Table 4: Durability of factors associated with LOS > 5 days by GLM following recalibration at weeks 1,4,8,12**

|  | Week 1 | | Week 4 | | Week 8 | | Week 12 | |
| --- | --- | --- | --- | --- | --- | --- | --- | --- |
|  | Coef | P value | Coef | P value | Coef | P value | Coef | P value |
| Nursing Home Admission | 1.340 | <0.001 | 1.079 | <0.001 | 1.099 | <0.001 | 1.033 | <0.001 |
| Inter-Hospital Transfer | 1.549 | <0.001 | 1.441 | <0.001 | 1.385 | <0.001 | 1.409 | <0.001 |
| No Oxygen Requirement | -1.521 | <0.001 | -1.391 | <0.001 | -1.348 | <0.001 | -1.324 | <0.001 |
| Mechanical Ventilation | 1.320 | 0.001 | 1.323 | <0.001 | 1.558 | <0.001 | 1.486 | <0.001 |
| Weight Loss | 0.808 | 0.006 | 0.622 | 0.020 | ------ | NS | 0.438 | 0.036 |
| Comorbidity Sum | ------ | NS | 0.063 | 0.045 | 0.120 | <0.001 | 0.100 | <0.001 |
| Diastolic Blood Pressure | ------ | NS | ------ | NS | -0.017 | 0.008 | ------ | NS |

**Table 5: Durability of factors associated with LOS > 10 days by GLM following recalibration at weeks 1,4,8,12**

|  | Week 1 | | Week 4 | | Week 8 | | Week 12 | |
| --- | --- | --- | --- | --- | --- | --- | --- | --- |
|  | Coef | P value | Coef | P value | Coef | P value | Coef | P value |
| Nursing Home Admission | ------ | NS | ------ | NS | 0.723 | 0.001 | 0.890 | <0.001 |
| Inter-Hospital Transfer | 0.925 | <0.001 | 0.930 | <0.001 | 0.801 | <0.001 | 1.045 | <0.001 |
| No Oxygen Requirement | -0.777 | 0.004 | -0.864 | 0.001 | -0.605 | 0.024 | ------ | NS |
| Mechanical Ventilation | 1.502 | <0.001 | 1.578 | <0.001 | 1.396 | <0.001 | 1.484 | <0.001 |
| Other Neurologic Conditions | 0.633 | 0.003 | 0.641 | 0.002 | 0.498 | 0.013 | 0.449 | 0.013 |
| Weight Loss | 1.029 | <0.001 | 1.012 | <0.001 | 0.779 | <0.001 | 0.838 | <0.001 |
| Maximum O2 Requirement | ------ | NS | ------ | NS | 0.007 | 0.017 | 0.012 | <0.001 |
| Obesity | ------ | NS | ------ | NS | 0.665 | 0.003 | 0.702 | 0.001 |

**Table 6: Durability of factors associated with LOS > 15 days by GLM following recalibration at weeks 1,4,8,12**

|  | Week 1 | | Week 4 | | Week 8 | | Week 12 | |
| --- | --- | --- | --- | --- | --- | --- | --- | --- |
|  | Coef | P value | Coef | P value | Coef | P value | Coef | P value |
| Nursing Home Admission | 1.001 | <0.001 | ------ | NS | ------ | NS | 0.944 | <0.001 |
| Inter-Hospital Transfer | 1.224 | <0.001 | 1.255 | <0.001 | 1.350 | <0.001 | 1.394 | <0.001 |
| Mechanical Ventilation | 1.939 | <0.001 | 1.720 | <0.001 | 1.484 | <0.001 | 1.625 | <0.001 |
| Weight Loss | 0.844 | 0.002 | 0.794 | 0.003 | 0.896 | <0.001 | 0.754 | 0.001 |
| Comorbidity Sum | ------ | NS | 0.087 | 0.010 | ------ | NS | ------ | NS |
| Maximum O2 Requirement | ------ | NS | ------ | NS | 0.010 | 0.004 | 0.010 | 0.001 |
| Obesity | ------ | NS | ------ | NS | 1.257 | 0.001 | 1.172 | <0.001 |

* The model was allowed to recalibrate on a weekly basis including new patients that had been discharged prior to extraction (occurring on Friday) This shows selected weeks demonstrating factors remained consistent through time.
